# Electrolytic cleaning as a biofilm control strategy on medical implants: mechanisms, evidence and translational perspectives

**DOI:** 10.3389/fmicb.2026.1867507

**Published:** 2026-06-22

**Authors:** Lluis Font-Vizcarra, Beatriz Sobrino-Díaz

**Affiliations:** 1 Department of Surgery and Surgical Specialties, Faculty of Medicine and Health Sciences, University of Barcelona, Barcelona, Spain; 2 Department of Trauma and Orthopaedics, Hospital Moises Broggi i General d' Hospitalet, Consorci Sanitari Integral, L'Hospitalet de Llobregat, Spain; 3 Department Infection Desease, Hospital Regional Universitario de Malaga, Málaga, Spain; 4 Instituto de Investigacion Biomedica de Malaga, Málaga, Spain

**Keywords:** biofilm, bubbles, electric field, electrocleaning, hydrogen, medical device, physical disruption

## Abstract

Biofilm formation on medical devices represents a critical challenge in microbiology and clinical practice, as structured bacterial communities embedded in an extracellular polymeric matrix exhibit marked tolerance to antimicrobial agents and host defenses. In orthopedic implant-associated infections, these properties significantly limit the efficacy of conventional strategies based on surgical debridement and systemic antibiotic therapy, particularly when biofilm is established on metallic surfaces. Electrical-based approaches have been investigated as alternative strategies to target biofilm, including the application of electric fields and direct electrical currents. These methods influence bacterial viability and biofilm integrity mainly through electrochemical mechanisms, such as local pH changes and the generation of reactive species. However, their mechanisms remain heterogeneous and clinical translation has been limited. Electrolytic cleaning represents a distinct mechanistic paradigm based on water electrolysis at the solid–liquid interface. Hydrogen evolution at the cathode leads to gas bubble formation, which can exert mechanical forces on the biofilm structure and promote detachment from the underlying surface. This bubble-mediated mechanism provides a complementary physical approach to biofilm disruption. This minireview summarizes the physicochemical basis of water electrolysis and critically analyses the available experimental evidence. Although current data remain largely preclinical, the recent development of medical devices based on this technology suggests that clinical translation may be increasingly feasible. Nevertheless, robust evidence in patients is still lacking, and further studies are required to define safety, efficacy and optimal application parameters in clinically relevant settings.

## Introduction: antimicrobial methods of electric fields

The antimicrobial potential of electric fields has been recognized for more than a century. Anderson and Finkelstein described as early as 1919 the use of low-frequency alternating current (AC) to treat milk (“electropure process”), effectively achieving microbial reduction in a food matrix (Anderson and Finkelstein, 1919). In other non-medical fields, electric fields—usually at high voltage—have long been incorporated into industrial water treatment as part of microbial control strategies (Alkhadra et al., 2022).

In the biomedical arena, both direct and alternating field modalities have been explored for controlling implant-associated biofilms. Direct current (DC) approaches include continuous low-amperage DC (from μA/cm^2^ to mA/cm^2^), high-amperage (from hundreds of mA/cm^2^ to A/cm^2^) pulsed fields and cathodic voltage-controlled stimulation (CVCES), while alternating approaches comprise alternating current (AC) electric fields, alternating magnetic fields (AMFs) and combined electric/magnetic schemes (Blenkinsopp et al., 1992; Costerton et al., 1994; Del Pozo et al., 2008, 2009a, b; Giladi et al., 2008). Direct current (DC) and alternating current (AC) induce distinct physicochemical effects. DC produces sustained electrode polarization and may promote electrochemical phenomena including electrolysis, local pH changes, reactive species generation and hydrogen evolution. Conversely, AC periodically reverses electrode polarity and, depending on frequency, may influence bacterial adhesion, membrane integrity or induce thermal effects while reducing persistent electrochemical gradients.

Nevertheless, the translational application of low-frequency AC systems to implant-associated infections may remain limited by electrochemical alterations at the electrode interface. In orthopedic titanium models, low-intensity alternating pulses capable of reducing bacterial adhesion were also associated with electrolysis, electrocoagulation phenomena and anodisation/oxidation of titanium surfaces, raising concerns regarding direct *in vivo* applicability (Bernaus et al., 2022).

Depending on whether current is applied, as well as on variations in potential difference or current intensity, electrode composition and polarity, and the physicochemical properties of the electrolyte, distinct mechanisms of microbial inactivation can be achieved:

### Enhanced antibiotic efficacy (the bioelectric effect)

In the combination of electrical stimulation with systemic antibiotics, Costerton and colleagues were pivotal: Blenkinsopp et al. showed that low-intensity electrical fields enhanced the activity of biocides against Pseudomonas aeruginosa biofilms, coining the concept of the “bioelectric effect” (Blenkinsopp et al., 1992) and Costerton et al. (1994) later explored the mechanisms underpinning this phenomenon. Del Pozo and Patel's group subsequently summarized this work in a systematic review and extended it to several *in vitro* and *in vivo* models, demonstrating that low-intensity electrical currents can inhibit growth and reduce biofilm burden of *S. aureus, P. aeruginosa and S. epidermidis* on biomaterials (Del Pozo et al., 2008, 2009b; Giladi et al., 2008). In a rabbit model of chronic foreign-body osteomyelitis due to *S. epidermidis*, they also showed that prolonged low-intensity direct current combined with antibiotics significantly reduced bacterial load in bone and on the implant (Del Pozo et al., 2009a).

### Induction heating

An alternating magnetic field generated by high-frequency alternating current (AC) induces eddy currents within the metallic implant (e.g., titanium, stainless steel), resulting in localized resistive heating capable of disrupting the adherent bacterial biofilm from metallic implants by causing rapid Joule heating to 55–90 °C on surfaces within 3–7 min (Wang et al., 2021). This localized hyperthermia lyses bacterial cells and disrupts extracellular polymeric substances (EPS) matrices in biofilms (*S. aureus, S. epidermidis*), achieving >5-log reductions (Chopra et al., 2017) of totally eradication when combined with vancomycin (Pijls et al., 2020). Post-surgical application via external coils may be a theoretical non-invasive adjunct for prosthetic infections, often synergising with antibiotics (Irmak Šalandová et al., 2025).

However, despite its potential advantages, the clinical translation of induction heating remains limited by the narrow balance between antibiofilm efficacy and thermal safety. Bone tissue is sensitive to heat, and classical experimental studies demonstrated that exposure to approximately 47 °C for 1 min may induce thermal injury, while higher temperatures produce progressively greater biological effects (Eriksson and Albrektsson, 1983). More recent orthopedic literature has confirmed that thermal osteonecrosis depends not only on peak temperature but also on exposure duration, irrigation, tissue perfusion and local heat dissipation (Augustin et al., 2008). Therefore, although induction heating may preferentially confine heat generation to metallic components and short exposure times may reduce heat diffusion to surrounding tissues, tissue safety cannot be assumed and requires careful control of time–temperature parameters.

An additional limitation relates to the potential for heterogeneous heat distribution across complex implant geometries. Orthopedic implants frequently present irregular shapes, variable thicknesses and multi-component metallic structures that may modify local electromagnetic coupling and heat generation. Consequently, thermal hot and cold spots may develop along the implant surface, potentially leading to uneven biofilm clearance. While insufficient heating may reduce antimicrobial efficacy, excessive local temperatures could increase the risk of thermal injury to surrounding tissues. This issue may be particularly relevant in large implants or complex reconstructions, where achieving homogeneous temperature distribution could be challenging.

**Figure 1:**
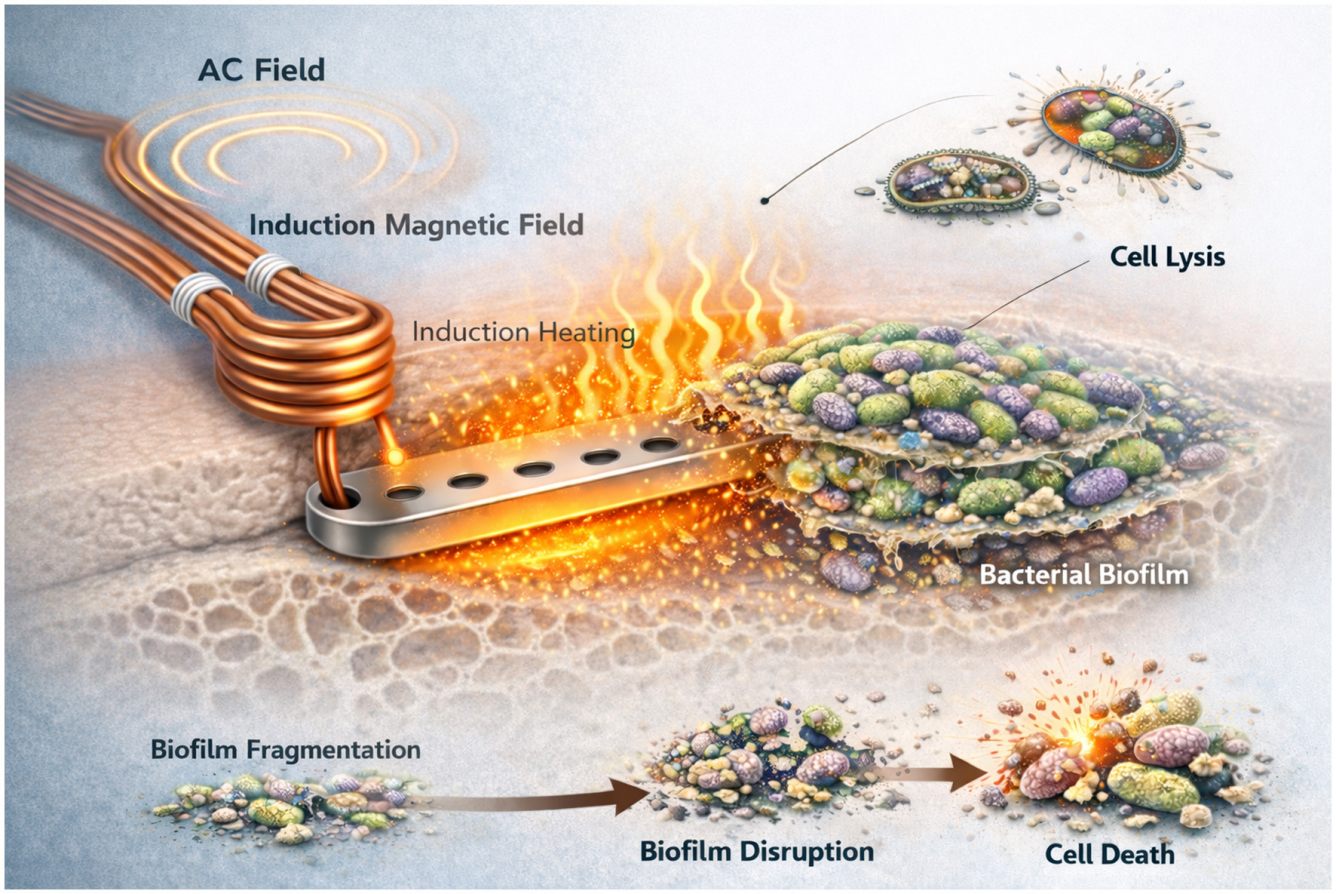
Induction-mediated thermal disruption of bacterial biofilm on an orthopedic implant. High-frequency alternating current generates an alternating magnetic field that induces eddy currents within the metallic osteosynthesis plate, resulting in localized heating. The controlled temperature rise disrupts the biofilm matrix, damages bacterial membranes and leads to cell lysis and biofilm fragmentation.

From a clinical perspective, transient thermal insults are routinely encountered during orthopedic procedures such as drilling, sawing and PMMA cement polymerization. However, these examples illustrate that clinical impact depends on the interaction between temperature, exposure duration and spatial heat distribution rather than temperature alone. Consequently, induction heating strategies require implant-specific validation and ideally thermal monitoring before clinical translation (Figure 1).

### Electrostatic disruption

It is well established that most bacteria exhibit a net negative surface charge (Wilson et al., 2001; Halder et al., 2015). Drawing on this principle, research has pursued electric fields to modulate surface polarity, thereby reducing bacterial adherence or inducing detachment of established biofilms (Poortinga et al., 2001). Experimental work has shown that electric fields can alter bacterial attachment forces to conductive surfaces (Gall et al., 2013; Poortinga et al., 2002; Muñoz-Mahamud et al., 2018), and that electric current can induce detachment and decreased viability of *S. epidermidis* biofilms on surgical stainless steel (van der Borden et al., 2004, 2005).

### Electrochemical reactions and generation of reactive species

Depending on electrode polarity, electrolyte composition (e.g., NaHCO_3_ or saline), and current direction, electrical fields may trigger redox reactions producing reactive oxygen species (ROS) (Brinkman et al., 2016), local pH shifts, or chlorine species (Liu et al., 1997), thereby inducing oxidative damage to bacterial cells and the extracellular polymeric substance (EPS). Anodic conditions are typically associated with oxidation reactions and may promote chlorine generation and/or local acidification, whereas cathodic conditions may induce oxygen reduction reactions leading to hydroxyl ion production, local pH elevation and H_2_O_2_ formation under specific conditions (Istanbullu et al., 2012). In parallel, and as described in the following section, cathodic setups may also generate hydrogen microbubbles through the Hydrogen Evolution Reaction (HER), providing a distinct physical mechanism potentially contributing to biofilm detachment and forming the basis of electrolytic cleaning approaches.

### Electrocleaning of metallic surfaces

The observation of bubbles in cathodic electrode underpin the concept of using electricity not only as an inductor of electrochemical changes or “drug enhancer” but also as a physical means of cleaning contaminated metal surfaces via electrolysis-driven gas formation at the interface, analogous to industrial electrolytic cleaning. An *in vitro* study from Bernaus et al. (2025) reported bacterial count reductions of up to 8 log were obtained, and when electrical pulses were combined sequentially with standard surgical pulse lavage, total reductions of up to 13 log compared with untreated controls were achieved. These data support a mechanistic model in which brief bursts of direct current induce local electrolysis at the metal–fluid interface, generating hydrogen bubbles that mechanically scour the implant surface and detach adherent bacteria and biofilm fragments.

## From water electrolysis to biofilm electrocleaning

**Figure 2:**
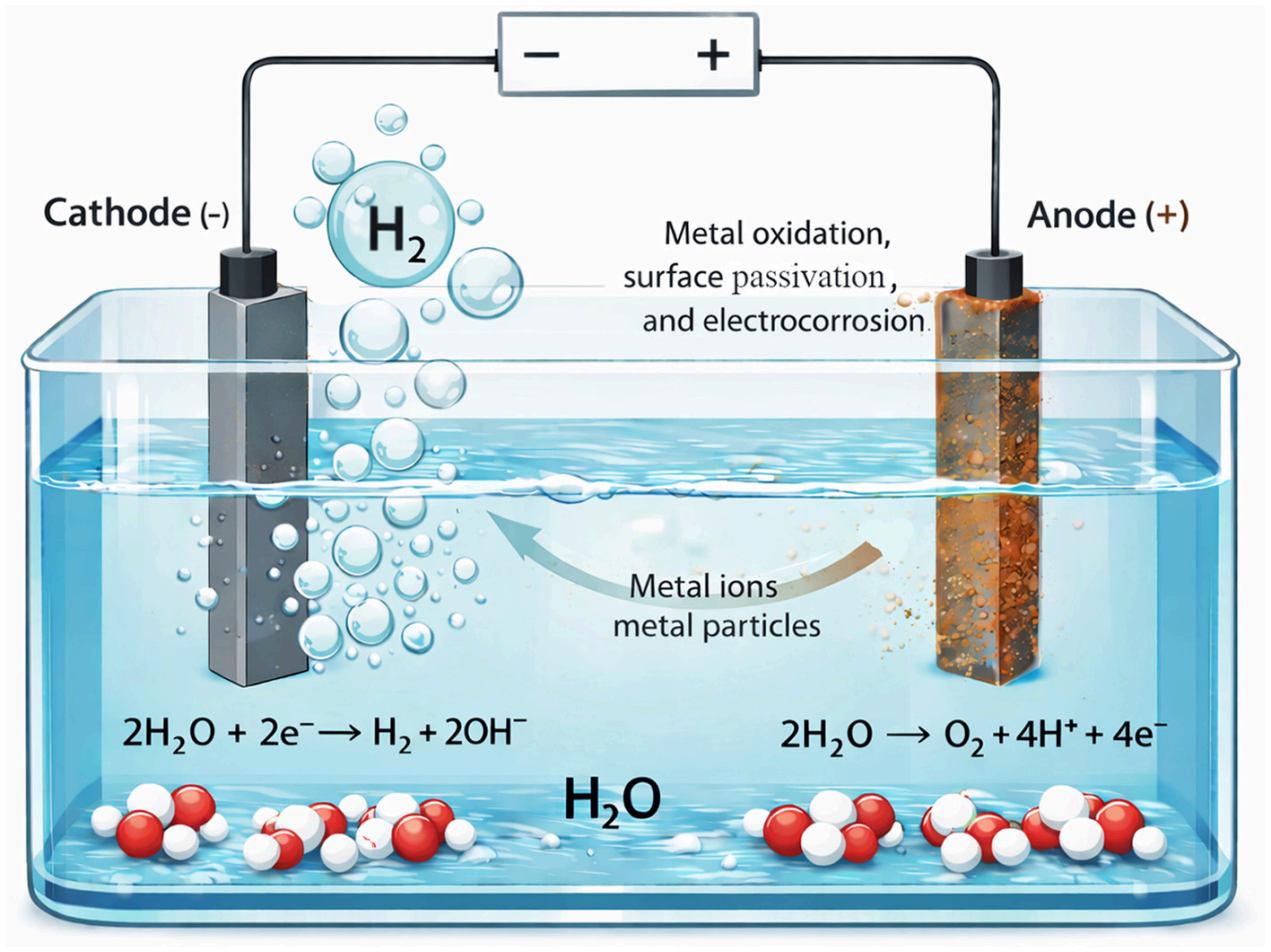
Schematic representation of water electrolysis when an electric current passes through an aqueous medium. At the cathode (negative electrode), water reduction generates hydrogen gas (H_2_) and hydroxide ions (OH^−^), while at the anode (positive electrode) water oxidation produces oxygen gas (O_2_) and protons (H^+^). Because pure water has very low electrical conductivity, electrolysis requires the presence of dissolved ions acting as an electrolyte. In experimental and clinical settings, saline solution (NaCl 0.9%) is commonly used. In the presence of chloride ions, additional reactions may occur, including the formation of chlorine gas (Cl_2_) at the anode and sodium hydroxide (NaOH) in the cathodic region. Following the invention of the voltaic pile by Alessandro Volta in 1800, Carlisle and Nicholson employed this continuous current source to decompose water into hydrogen and oxygen in a controlled and reproducible manner. In the same year, Ritter independently performed comparable experiments in Jena, Germany, further consolidating the experimental basis of water electrolysis. Shortly thereafter, Cruickshank extended these observations to saline solutions, demonstrating the electrolytic decomposition of sodium chloride into hydrogen and chlorine. Despite these seminal discoveries, several decades elapsed before such processes were translated into practical technical applications.

Electrolysis or water decomposition by electricity (Figure 2) has been known for more than two centuries and constitutes the historical foundation of modern electrolytic cleaning. The first documented generation of hydrogen gas by electrical means dates back to 1789, when van Troostwijk and Deiman successfully decomposed water using an electrostatic generator as a direct current source (Smolinka et al., 2022).

As electrolytic methods were progressively adopted in industrial settings, it became evident that hydrogen evolution at the cathodic surface was not merely a chemical by-product, but also a physical agent capable of exerting mechanical forces at the solid–liquid interface. The nucleation, growth and detachment of hydrogen bubbles were observed to remove grease, oxides and adherent contaminants from metallic substrates (Sun et al., 2017), thereby establishing the conceptual basis of electrolytic cleaning methods based predominantly on gas-mediated mechanical detachment rather than on chemical dissolution.

This physical cleaning effect, well recognized in industrial surface treatment, later provided a mechanistic framework for understanding how electrolytic processes could be applied to biologically contaminated surfaces (Kumar et al., 2012). When an electric current is applied in an aqueous environment, hydrogen bubbles form directly at the surface of the conductive material. As these bubbles grow, coalesce and detach, they generate local shear forces and micro-currents that act tangentially to the surface. This dynamic process disrupts weak adhesive forces and mechanically lifts adhered biofilms, in a manner analogous to the detachment of dirt during industrial electrolytic degreasing.

Bacterial biofilms share key physical characteristics with industrial fouling layers: they are hydrated, viscoelastic structures anchored to surfaces through a combination of electrostatic interactions, extracellular polymeric substances and surface irregularities. The mechanical action generated by hydrogen bubble evolution directly targets this mode of attachment. Bubble expansion beneath and within the biofilm matrix produces localized deformation and peeling forces, leading to fragmentation and detachment of the biofilm.

Importantly, this mechanism is fundamentally physical in nature. The detachment of biofilm occurs as a consequence of bubble-induced mechanical stress and interface disruption, rather than through the generation of cytotoxic chemical species such it happens in electrochemical methods for controlling biofilm. This distinction is particularly relevant in biomedical contexts, where preservation of the underlying material and avoidance of chemical damage are essential. This non-pharmacological strategy offers a conceptually simple yet biologically effective approach to disrupting surface-adherent microbial communities.

## *In vitro* evidence

The first direct experimental reference explicitly identifying hydrogen bubbles generated by electrolysis as a mechanism for bacterial biofilm detachment is generally attributed to Gião and colleagues (Gião et al., 2005), who demonstrated that hydrogen bubble formation at a negatively polarized electrode could physically lift *Pseudomonas fluorescens* biofilms from platinum surfaces. In this early work, biofilm removal was clearly associated with gas evolution rather than with bactericidal chemical effects.

Shortly thereafter, Rabinovitch and Stewart (2006) provided a simple and highly illustrative experimental confirmation of this phenomenon using a 6-V battery connected to stainless steel coupons bearing *S. epidermidis* biofilms. In their original report, the authors described a rapid detachment of biofilm within approximately 30 s at the electrode they referred to as the anode, coinciding with visible gas bubble formation. However, as later acknowledged by the authors themselves, the terms “anode” and “cathode” had been inadvertently interchanged in the original publication. A formal correction published 11 years later (Rabinovitch and Stewart, 2017) clarified that the rapid bubble-mediated biofilm detachment occurred at the cathode, consistent with hydrogen evolution, while the opposite electrode was associated primarily with oxidative effects.

In a well-cited *in vitro* study, Schneider et al. (2018) systematically evaluated the electrochemical removal of bacterial biofilms from titanium surfaces, demonstrating that electrically induced surface phenomena could disrupt mature biofilms without mechanical instrumentation. Although multiple physicochemical factors were analyzed, the authors highlighted the relevance of cathodic reactions and surface gas formation in weakening biofilm adhesion on implant-grade titanium.

Further *in vitro* evidence was provided by (Ratka et al. 2019) who compared electrolytic cleaning with conventional mechanical debridement on biofilm-contaminated implant surfaces. Their laboratory model showed that electrolytic treatment achieved near-complete biofilm removal on complex surface topographies, including threaded and roughened areas, which are typically resistant to mechanical methods. The authors explicitly associated this effect with hydrogen bubble formation at the implant surface, facilitating mechanical lifting of the biofilm matrix.

More recent *in vitro* comparative studies have focused both on optimizing electrolytic parameters and on benchmarking electrolytic cleaning against alternative decontamination techniques. Assunção et al. (2023) demonstrated that electrolytic methods produced greater biofilm reduction on titanium discs than brushes or powder-based systems, particularly within surface micro-irregularities. Scanning electron microscopy revealed extensive structural disruption and detachment of the biofilm consistent with bubble-mediated lifting rather than abrasive surface damage. In a further comparative investigation, Zhu et al. (2024) evaluated electrolytic cleaning against five different decontamination approaches in a multi-species biofilm model. Beyond analyzing the influence of current intensity and exposure time, the authors directly compared biofilm removal efficacy and surface alterations across methods. Electrolytic cleaning achieved the most pronounced biofilm reduction while preserving the microtopography of the titanium Surface and maintaining favorable characteristics associated with osteogenic potential. By contrast, certain mechanical techniques showed either incomplete biofilm removal or detectable surface modification. Together, these findings reinforce the concept that cathodic hydrogen bubble formation enables effective biofilm detachment on rough and microstructured titanium surfaces while remaining comparatively surface-friendly.

Finally, *in vitro* multi-species biofilm models have expanded the relevance of hydrogen bubble-mediated detachment beyond single-organism systems. Virto et al. (2023) and Zhu et al. (2024) independently showed that electrolytic cleaning significantly reduced multi-species biofilms grown on sandblasted and acid-etched titanium, with efficacy closely linked to current density and treatment duration. Both studies emphasized that the negative electrode acted as the primary site of biofilm removal, reinforcing the concept that cathodic hydrogen evolution is central to the electrolytic detachment mechanism.

**Figure 3:**
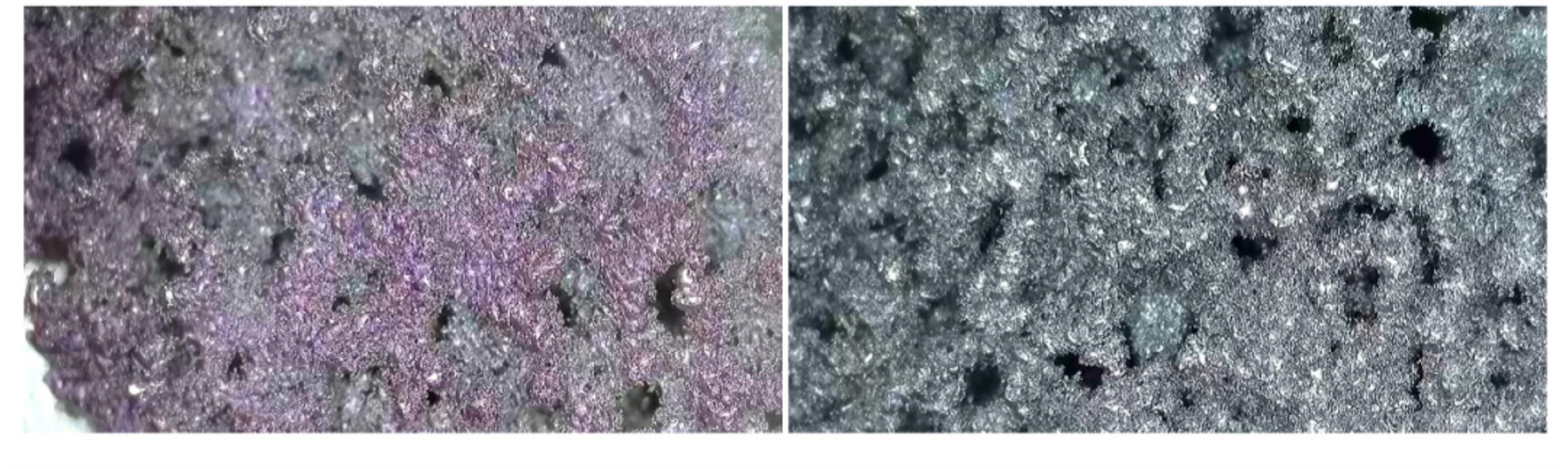
Illustrates the macroscopic appearance 10 × magnification view of porous titanium plates uniformly coated with an *Escherichia coli* biofilm stained with crystal violet, before (left picture) and after (right picture) electrolytic treatment. The electrolytic bath consisted of 30 ml of 0.9% saline solution, applied for 10 s at a current intensity of 1 A, using pulsed delivery at 4 pulses per second with a 50% duty cycle. Following treatment, complete disappearance of adherent biofilm residues was observed, both macroscopically and under magnification, confirming the ability of hydrogen bubble-mediated detachment to effectively clean complex and porous titanium surfaces.

The physical action of hydrogen bubble formation enables electrolytic cleaning to act on any surface area reached by the electrolyte, irrespective of surface finish. As the mechanism relies on bubble nucleation, growth and detachment at the solid–liquid interface, its efficacy is not limited to smooth or polished materials. Consequently, electrolytic cleaning remains effective on roughened, non-polished and even porous surfaces, provided that electrolyte penetration is achieved (Figure 3).

## *In vivo* and clinical evidence

With regard to *in vivo* evidence, the data that most directly evaluate an electrolytic cleaning process involving gas bubble generation, consistent with a cathodic hydrogen (H_2_) mechanism, originate predominantly from the field of dental implantology. In a canine peri-implantitis model, histological findings have demonstrated the possibility of re-osseointegration following decontamination of infected implants using an electrolytic bubbling-based procedure (Schlee et al., 2020). These observations support the concept that a physically driven mechanism may act on adherent biofilm even within a complex biological environment, although such findings should not be directly extrapolated to orthopedic prosthetic surgery without caution.

Beyond animal models, clinical evidence in humans has been generated using a commercially available device (GalvoSurge^®^), whose mechanism of action is explicitly described as electrolytic cleaning through hydrogen bubble formation leading to biofilm detachment. A randomized controlled clinical trial evaluating this approach in the surgical–regenerative treatment of peri-implantitis reported favorable short-term outcomes when electrolytic cleaning was incorporated into the protocol, compared with conventional mechanical decontamination (Schlee et al., 2019). Importantly, follow-up of the same cohort at 18 months supports maintenance of favorable clinical and radiographic outcomes over the medium term (Schlee et al., 2021). These clinical data provide proof-of-principle that electrolytic biofilm detachment can be safely applied *in vivo* and may contribute to improved local biological conditions following implant decontamination.

Additional insight into the biological effects of electrolytic cleaning has been provided by human histological evaluations following delayed explantation. In these studies, histomorphometric analysis demonstrated evidence of re-osseointegration after electrolytic cleaning and regenerative therapy, offering rare direct information on tissue behavior and surface decontamination at the bone–implant interface in humans (Bosshardt et al., 2022). Although limited in scale, such observations strengthen the biological plausibility of this approach.

From an orthopedic perspective, animal models of implant-associated infection treated with cathodic electrical stimulation have also been described. In these studies, local electrical stimulation applied to metallic implants resulted in reduced bacterial burden in experimental settings (Ehrensberger et al., 2015; Weeks et al., 2023; Cavanaugh et al., 2016). While these investigations involve electrochemical processes in which local electrolysis and gas formation may occur, they were not designed to isolate the specific contribution of hydrogen bubble-mediated biofilm detachment. Consequently, they should be interpreted as indirect supportive evidence of the biological safety and antibacterial potential of applying local electrical energy to metallic implant surfaces, rather than as direct demonstrations of an electrolytic cleaning mechanism.

Taken together, the available *in vivo* and clinical data suggest that electrolytic cleaning based on cathodic hydrogen bubble generation is biologically feasible and clinically applicable, at least within the dental implant field. However, the extent to which these findings can be translated to orthopedic implant infections remains to be established and warrants further dedicated clinical investigation.

## Potential orthopedic applications and translational considerations

**Figure 4:**
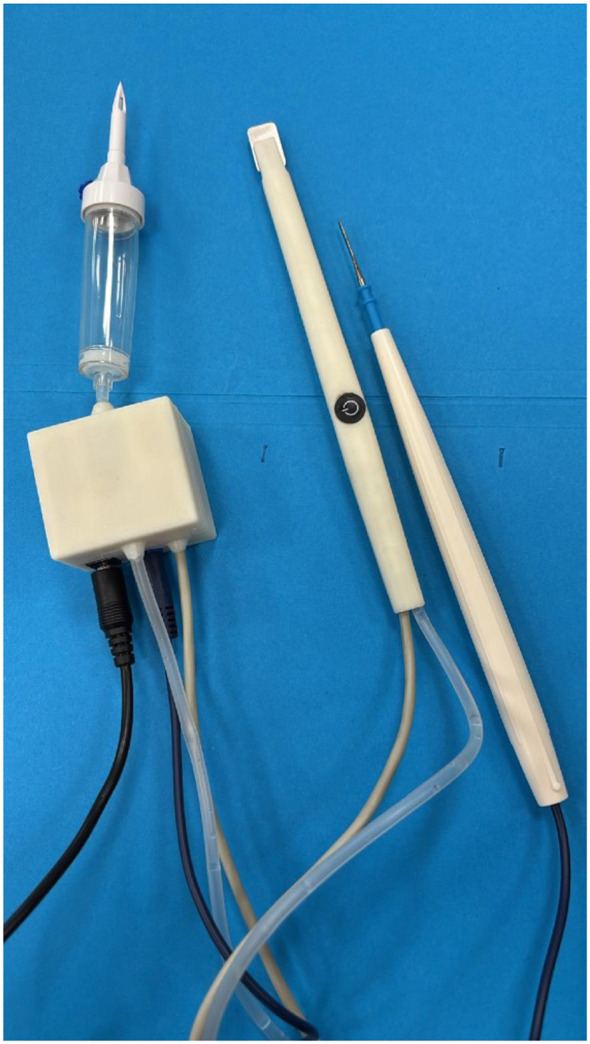
Single-use electrolytic cleaning device. Photograph of the single-use device designed for intraoperative electrolytic cleaning of metallic implants. The implant is polarized as the cathode through contact with the negative pole (electrosurgical tip), while the opposite hand-piece contains the positive electrode surrounded by a sponge delivering the electrolyte. Sweeping the electrode across the implant surface generates hydrogen bubbles as a result of water electrolysis.

The available mechanistic and preclinical data provide a rationale for cautiously exploring electrolytic cleaning as an adjunctive intraoperative strategy in orthopedic implant surgery. A single-use device has been developed to enable controlled cathodic hydrogen bubble-mediated surface treatment of metallic prosthetic components (Figure 4). This technology is the result of more than 15 years of research conducted by a multidisciplinary group of Spanish investigators in collaboration with an industrial consortium. The primary therapeutic context in which such a technology may be considered is debridement, antibiotics and implant retention (DAIR) for acute periprosthetic joint infection. In this setting, the principal challenge is disruption of mature surface-adherent biofilm. Electrolytic cleaning does not aim to replace established surgical principles or systemic antimicrobial therapy, but rather to provide an additional physical mechanism of biofilm detachment that may complement mechanical debridement.

An important practical consideration is that electrolytic cleaning is inherently limited to surfaces that can be electrically addressed, namely the exposed metallic components of the implant. It does not directly target contaminated peri-implant soft tissues, necrotic debris or infected bone surfaces, which remain dependent on meticulous surgical debridement. In this context, the use of chemical irrigation solutions retains a central role. It is therefore relevant to distinguish between their use in infection prevention and in the treatment of established infection. In primary arthroplasty and other clean orthopedic procedures, dilute povidone-iodine lavage is widely employed immediately prior to wound closure with the intention of reducing early bacterial contamination, with clinical studies suggesting a reduction in postoperative infection rates compared with saline alone, albeit with some heterogeneity in outcomes (Kim et al., 2020; Kobayashi et al., 2021; Shohat et al., 2022). Conversely, during DAIR procedures, irrigation solutions specifically formulated to disrupt biofilm structure, such as surfactant- and chelator-based formulations (e.g., Bactisure^®^), have been proposed and are currently being investigated as adjuncts to standard lavage (Oleo-Taltavull et al., 2024).

Although the underlying principles of electrolysis and electrically induced biofilm disruption have been described in experimental settings for several decades, their translation to orthopedic surgery has been hindered. This is likely related to the specific technical challenges associated with large metallic implants, including the need to control current delivery over extended surfaces, minimize thermal and electrochemical risks to surrounding tissues, and achieve effective bubble-mediated cleaning while maintaining procedural simplicity. Addressing these constraints requires careful optimization of electrical parameters and device configuration, as well as the development of practical, intraoperatively applicable systems. In this context, the present approach represents an attempt to bridge the gap between established electrochemical principles and their safe and reproducible application in orthopedic surgery.

Within this framework, electrolytic cleaning may be conceptually positioned as a surface-specific adjunct targeting the implant itself, while chemical irrigation and surgical debridement address the surrounding biological environment. Rather than representing alternative strategies, these approaches may be complementary: physical detachment of biofilm from metallic surfaces could be combined with chemical disruption and removal of residual contamination in peri-implant tissues. In addition, a potential preventive application may be considered, whereby brief electrolytic treatment is applied immediately before closure in prolonged procedures or in cases involving large tumor implants with an increased risk of intraoperative bacterial contamination. Both therapeutic and preventive roles remain investigational and require formal clinical validation. From a translational standpoint, the device has been applied experimentally in selected human cases under last-resort or compassionate-use conditions, providing preliminary insights into feasibility and short-term safety. Early-phase clinical studies are ongoing, although robust orthopedic clinical evidence is still limited and conclusions regarding efficacy must remain cautious. If validated in appropriately designed clinical studies, this approach could contribute to improving implant preservation strategies and expanding the therapeutic options available for the management of implant-associated infections.
